# For the Community, by the Community: Advancing Research for Latino Communities in Dementia Care Following the Guidance of Latino Older Adults

**DOI:** 10.5888/pcd22.240381

**Published:** 2025-05-22

**Authors:** Maria Mora Pinzon, Susana Fernandez de Cordova, Valentina Flores Diaz, Maria Del Carmen Rosales, Ramon Argandoña, Nilda Bermudez, Beatriz Caro, Tomas Caro, Maria Cristina Martinez, Yolanda Salazar, Gina Green-Harris, Kat Phelps

**Affiliations:** 1 Department of Medicine, Division of Geriatrics and Gerontology, School of Medicine and Public Health, University of Wisconsin — Madison; 2 Wisconsin Alzheimer’s Institute, School of Medicine and Public Health, University of Wisconsin — Madison; 3 Latino Community Advisory Board for Healthy Aging, Madison, Wisconsin; 4 Center for Community Engagement and Health Partnerships, School of Medicine and Public Health, University of Wisconsin — Madison; 5 Wisconsin Network for Research Support, School of Nursing, University of Wisconsin — Madison

## Abstract

We outline a practical approach to engaging Latino communities in dementia care research by establishing a community advisory board (CAB). Acknowledging the historical underrepresentation of Latinos in health research, we aimed to ensure that Latino perspectives and concerns were central to our research endeavors. As an initial step we adapted the *Patient Advisor Toolkit 1: Orientation for Patient Advisory Committees (PAT-1)* created by the Wisconsin Network for Research Support at the University of Wisconsin — Madison School of Nursing to the needs of Latino/a/e/x populations. We describe the CAB formation process, from initial outreach to community members and organizations to the recruitment, engagement, and retention of CAB members. CAB members provided guidance on the legibility and clarity of study materials and assisted with strategic planning for outreach initiatives related to healthy aging, brain health, and dementia risk reduction. Our enduring connections with CAB members and community partners have ensured that our research activities address community needs, provide benefits to the community, and inform our approach to connecting with Latinos in a culturally appropriate way. To foster successful CAB engagement and retention, we recommend 1) building trust with partners, 2) establishing clear expectations, 3) ensuring inclusive communication formats, 4) maintaining open communication, 5) offering honoraria and recognition, 6) affirming CAB members’ contributions, 7) understanding power dynamics between the academic institution and the community, and 8) ensuring adequate staff and programmatic support. This approach fosters trust-based relationships and meaningful engagement that advances health equity.

**Summary**

**What is already known on this topic?**

Community advisory boards are crucial to ensure that community preferences and needs are accounted for in research studies involving underresourced populations such as Latino communities.

**What is added by this report?**

We provide specific examples of how to engage Latino older adults in a community advisory board using diverse methods of communication to ensure inclusive participation in meetings. We describe the processes that have resulted in a collaboration that spans multiple projects.

**What are the implications for public health practice?**

The strategies outlined in this report can ensure that public health efforts are culturally relevant, equitable, and have a lasting impact on health equity.

## Background

Latino/a/e/x individuals represent 18% of the US population but less than 1% of the participants in clinical trials for Alzheimer’s disease and related dementia (ADRD) (1). The underrepresentation of Latino/a/e/x populations in clinical research limits our ability to effectively address health disparities among the fastest-growing demographic in the US. Meaningful progress in ADRD research requires the engagement of Latino/a/e/x communities in research to gain a deep understanding of the community’s needs, leverage existing resources, and develop innovative, sustainable solutions to improve health equity for all (2,3).

Community engagement is usually described as a continuum, with varying levels of involvement and collaboration that include informing, consulting, involving, collaborating with, and empowering the community (4). This progression reflects the deepening of relationships and increasing levels of shared leadership, demonstrating flexibility for partners to decide in which level of work or engagement they wish to invest. For some communities, shared leadership is the goal, while for others, an advisory role is preferable (5). Fundamental principles of community engagement include being clear about goals, understanding the community, building trust, partnering with the community, respecting diversity, providing information and feedback, being flexible and adaptable, and committing to long-term engagement. These principles ensure that the engagement is meaningful, respectful, and beneficial for all parties involved. Similarly, community-based participatory research (CBPR) (2,3,6) refers to a specific approach to research that involves community members as equal partners in all aspects of the research process. Although these are similar concepts, not all community engagement is CBPR, but all CBPR should include community engagement.

Implementing and sustaining community engagement and CBPR requires overcoming various challenges, including lack of resources, institutional constraints, lack of trust, power dynamics, and finding partners from community organizations (2,3,7). The development of infrastructure, such as community advisory boards (CABs), to engage the community and community organizations can help to overcome such challenges. CABs help formalize collaborations with academic institutions and researchers to provide action steps that can be implemented to dismantle barriers to engagement and research participation (8,9).

CABs are defined as groups of community representatives assembled to provide guidance and feedback on various aspects of research studies or programs. CABs typically consist of people with common interests, experiences, or cultural backgrounds related to the research topic (10). They serve as a bridge between researchers and the community, ensuring that research is culturally appropriate, ethically sound, and relevant to community needs (11). While CABs are increasingly recognized as valuable components of CBPR, there is limited information on how to adapt current recommendations for older Latino communities; in particular, with regard to member selection and recruitment, strategies for reducing barriers to participation, practical methods for establishing shared expectations, and approaches to maintaining engagement (11). We describe processes to establish a CAB of Latino/a/e/x older adults to ensure that Latino voices and priorities drive research efforts and produce culturally relevant approaches. In this article, the term Latino/a/e/x is intentionally used to reflect the range of identities within the Latin American community and might be used interchangeably with the word Latino.

## Community Advisory Board Goals and Ideal Membership

The main criteria for becoming an advisor were identifying as Latino/a/e/x or Hispanic, being an older adult or caregiver, and being willing to share ideas and opinions with the University of Wisconsin — Madison researchers. CAB members were not required to have prior experience related to ADRD; this decision was a result of ADRD being underdiagnosed in the Latino community. Less than 50% of those with the condition receive a diagnosis (12); thus, requiring experience based on a formal diagnosis would have limited participation of people who otherwise have the lived experience.

## Recruitment and Formation

We initiated this process through strategic outreach to local community-based organizations (CBOs) with established credibility (13) and extensive experience serving the Latino population in Madison, Wisconsin: 1) NewBridge, a senior center with over 25 years of experience in Latino senior programming and 2) The Latino Health Council of Dane County, an umbrella organization facilitating collaboration among Latino-focused services.

We implemented a multifaceted engagement strategy emphasizing learning from and with the community to deepen our understanding of Latino/a/e/x needs and challenges (2). Since 2018, we have actively participated in events hosted by the CBOs (eg, health fairs, educational lectures), co-hosted educational workshops focused on health issues, and had ongoing consultations with CBO leadership about incoming initiatives or research projects. These engagement efforts enhanced our cultural competence and demonstrated our commitment to the community’s well-being beyond our research objectives.

After these foundational relationships were firmly established, we created the CAB. This deliberate sequencing aligns with best community engagement practices, emphasizing building trust before formal collaborations (14). The CBOs played a pivotal role in helping us identify potential CAB members who reflected the diversity of the local Latino population and possessed the community insights needed to serve on the board. Through discussions with these CBOs about forming the CAB, we were introduced to a highly respected community leader with 25 years of experience developing programs and services for Latino adults, who became the first advisor of this CAB. This community leader identified and invited 4 well-connected community people who were known for providing candid opinions and constructive feedback. Additionally, the research team shared a flyer through community groups, which helped to identify and recruit another person who served as a caregiver for someone with dementia.

The recruiting activities took approximately 4 months, and the resulting CAB comprised 6 advisors from diverse Latino nationalities, ranging in age from late 60s to early 80s. The board’s composition was intentionally varied, including retirees, community activists, and former educators, ensuring a broad spectrum of experiences and perspectives.

## Meeting Planning

With the CAB membership established, the focus shifted to thoughtfully planning the first meeting to ensure that it was effective and inclusive. The first step was individually consulting with the advisors regarding their meeting format preferences. This consultation served multiple purposes: it addressed potential safety concerns related to the COVID-19 pandemic, acknowledged the transportation limitations faced by some members that could hinder consistent attendance at in-person meetings, and demonstrated the team’s commitment to inclusive decision making from the outset. We presented options for meeting formats, including in-person gatherings with safety protocols, hybrid meetings, and entirely virtual sessions. Unanimously, the advisors preferred virtual meetings, a choice that aligned well with the prevailing public health recommendations at the time that were related to the COVID-19 pandemic and addressed both safety and accessibility concerns. To ensure that all the advisors were present, the first meeting was set up approximately 2 months after membership was defined; this was a result of advisor’s limited availability because of travel or work commitments.

Recognizing the wealth of information to be shared and the importance of thorough engagement, we used the *Patient Advisor Toolkit 1: Orientation for Patient Advisory Committees (PAT-1)* (15) to guide the engagement strategies for the initial meetings. Developed by the Wisconsin Network for Research Support (WINRS) at the University of Wisconsin — Madison School of Nursing, this toolkit is designed to orient new patient advisors to their roles on advisory committees. PAT-1 facilitates the creation of tailored orientation sessions for community and patient advisors, a crucial step in establishing effective advisory meetings (15). This approach ensures that community members are adequately prepared for meaningful participation in research advisory roles, ensuring a solid foundation for collaborative engagement.

In adapting the PAT-1 toolkit for the Latino community, several vital modifications were made to ensure cultural and linguistic relevance. The toolkit was partially translated into Spanish, and the language was simplified to a high school reading level to enhance accessibility. Activities were modified to better align with cultural norms and expectations, such as omitting detailed explanations about institutional review boards and related compliance activities that might be unfamiliar or overwhelming to community members. The activities described in the PAT-1 toolkit as recommended for the initial meeting were spread across 2 meetings to accommodate a more relaxed pace and allow for ample discussion, providing advisors with additional time for questions and comments. This was key to building a shared understanding among advisors and fostering an environment where everyone felt comfortable contributing. Collaboration with experts from WINRS was maintained throughout the adaptation process to ensure that the modified activities remained consistent with the toolkit’s original goals while being culturally appropriate for Latino participants.

## Initial Meeting and Initial Agreement

The inaugural CAB meeting was held in July 2022 and was a 2-hour session formatted following the principles of the PAT-1 toolkit. It laid the foundation for collaborative work and set the tone for future interactions. Some of the topics discussed in that meeting:

- **Decision-making.** The group unanimously agreed to seek consensus among themselves, with the understanding that they would have an opportunity to ask questions of the principal investigator and research team for clarification or further inquiry about other options or opportunities.
- **Meeting planning.** Selecting a virtual meeting format required a more nuanced and detailed planning process compared with traditional in-person meetings. CAB members were asked about their preference for organizing and defining future meetings and were provided with some ideas, including 1) advisors defining the agenda and sharing it with the research team, 2) the research team organizing the agenda, or 3) a co-design process at each meeting. The consensus of the CAB was that the last 10 minutes of each meeting would be used to co-design the agenda for the following meeting. Furthermore, it was agreed that the research team would print and personally distribute meeting agendas, presentation materials, and discussion documents at least 1 week before each meeting to ensure all advisors had sufficient time to review and engage with the materials regardless of their technologic proficiency or access.
- **Scope of the work.** Advisors had the following questions about the work: *Will you provide services to the community? Can this group decide on the allocation of funds? Can this group develop new initiatives?* After a discussion on the limitations of funding and resources (eg, not being able to provide direct services, limited funding for activities with the intention of seeking additional funding for research that meets the community’s needs), the CAB members decided that there was still a benefit to participating. They saw this as an opportunity to learn about ADRD in a way that they could share with others while also providing their thoughts on what would be effective strategies and benefits for future research projects.
- **Terminology used by the CAB to describe the scope.** Because ADRD is considered part of normal aging in the Latino community, it was decided that using terms about health, prevention, and healthy aging to describe the group would be better accepted by the community. This terminology would also help the group to have a broader impact, by not only talking about topics of interest to those that have a diagnosis but also talking to everyone that might be at risk or caring for someone at risk.

An essential activity in the initial meeting was to review the flyer previously used to recruit CAB members. Advisors were encouraged to provide candid feedback on the flyer’s content, design, and overall message. This exercise allowed advisors to reflect on their recruitment experience and offer insights into the effectiveness of the outreach strategies, and it allowed the research team to assess the alignment between initial communications goals and the congruency with the experience of serving on the CAB. This review process served as a practical exercise in collaborative decision making, helping to build trust and establish a culture of open communication within the CAB.

## Functioning of the Community Advisory Board

As of July 2024, the CAB had met 14 times, approximately every 2 to 4 months, depending on the availability of the advisors. One of these meetings was an in-person celebration; there were 2 hybrid meetings (a mix of in-person and virtual attendance), and the rest were virtual. All meetings were conducted in Spanish because this was the preferred language of all CAB members. The Figure shows the process of convening and facilitating the meetings, with the roles of the people involved. All advisors were compensated for their time and expertise. The rate changed depending on the project (because of different funding sources) and varied between $50 and $75 per hour of meeting. This rate is on par with market rates (16) and did not include additional stipends provided, such as transportation or childcare expenses, which were addressed on a case-by-case basis.

**Figure:**
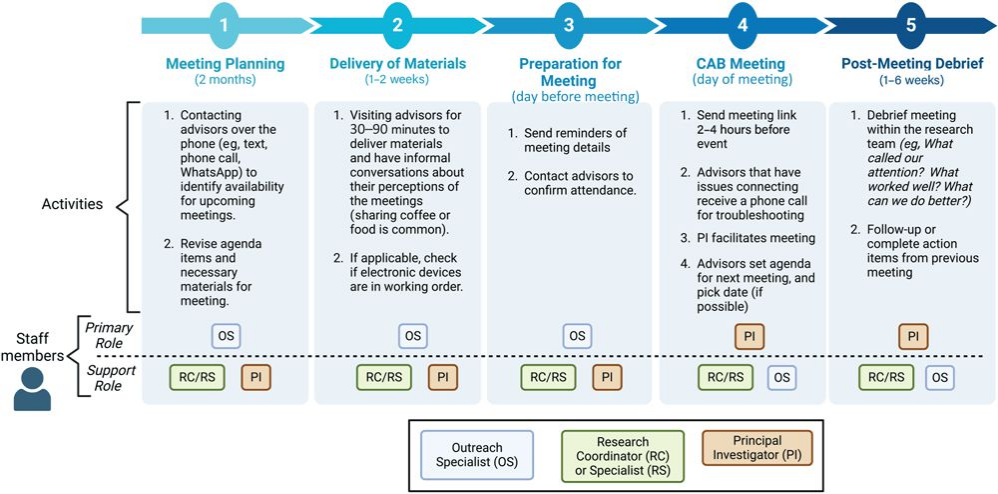
The process of scheduling and facilitating meetings of the community advisory board (CAB) for dementia care in Latino communities, Madison, Wisconsin.

To maintain a strong rapport and collaborative dynamic, we prioritized ongoing communication and personal interactions outside meetings, including sending small gifts or cards for their birthdays and holidays. A cornerstone of the engagement has been the delivery of printed materials to members’ homes before each meeting (as agreed). These interactions allowed the research team to address any questions or concerns the advisors had about upcoming meetings or materials, fostering open communication and strengthening personal connections with CAB members. Through these interactions, we identified instances of lack of clarity on their role as advisors, which originated from a perception that their recommendations had no value within the research or community engagement process. These were addressed at the time of those conversations and in subsequent meetings.

One aspect that was underestimated when the virtual format was decided was the need for a proactive and personalized approach to technological support. Research staff conducted additional in-home visits to CAB members who expressed difficulties with the virtual meeting platform to ensure that advisors’ computers were equipped with the necessary software for seamless participation in virtual sessions, and we provided support over the phone on the day of the meeting. Additionally, to support consistent participation, maintain inclusivity, and ensure all voices are heard and valued in the research process (17), the research staff established a multichannel reminder system that included email reminders 1 week prior and on the day of meetings, and shared meeting links via WhatsApp, a platform many members were comfortable using.

The CAB’s tasks and impact varied according to the project (Table 1). Overall, the principal investigator presented projects or initiatives to the CAB related to healthy aging and reducing risk factors for Alzheimer’s disease or other dementias. The CAB decided by consensus what topics or projects to get involved in. When feedback about specific materials was requested, the process was structured to ensure that we gathered their thoughts about relevance (which includes cultural relevance), readability, and usefulness. For our work, cultural relevance refers to ensuring that the information provided is relatable to Latinos by displaying or acknowledging traditions (eg, Dia de los Muertos), values (eg, family or community), or experiences (migration stories).

**Table 1:** Role and Recommendations Provided by Community Advisory Board (CAB) Members According to Project and Impact of Research Activities for Latino Communities in Madison, Wisconsin

Project title	Description of role of CAB members or tasks performed by the CAB	Impact
**Recordando Juntos en Salud — dementia outreach initiative**	**Guidance and feedback:** provided input on outreach strategies, indicating which places and conferences were the proper places for doing outreach. For example, the CAB said that participating in community events such as independence celebrations or other events not related to health topics was not useful because people do not attend these events to receive this information. The CAB advised that when discussing topics such as Alzheimer’s disease and related dementia it is better to create events for small groups or personalized activities that help to erase stigma around the condition. The CAB also emphasized the importance of education about mental health to raise awareness. **Outreach and engagement:** advisors participated in community events, sharing resources, and raising awareness about dementia and available support in the community.	Participated in the *“Ayudando con Cariño”* Conference in Waukesha, which was focused on caregivers for people with dementia, for 2 consecutive years and reached approximately 100 people. The research staff offered professional talks and educational content in Spanish. Participated in a local health fair for 2 consecutive years, providing services such as memory screenings and A_1c_ tests and sharing educational materials, reaching over 200 people. Collaborated with 2 local Latino nonprofit organizations serving caregivers. After listening to their needs, the research staff agreed to implement a 6-week stress management workshop for community members. Additionally, 6 health talks were conducted, focusing on brain and mental health and offering practical, evidence-based strategies for daily life. Overall, these activities reached over 150 people. Created content for social media on the main topics identified as a need, which has been seen by 15,712 people between July 1, 2023, and December 13, 2024.
**Brain Health Community Registry**	**Guidance and feedback:** provided feedback on the recruitment and intake materials to ensure that they were engaging and that the information on them was appropriate. The CAB also reviewed the intake form and went through all the demographic questions looking at language, cultural appropriateness, and length. A notable example was their input on questions related to sex and sexual orientation. While CAB members did not fully understand these questions, they did not find them offensive. However, they emphasized that such questions should be clearly marked as optional to avoid participants’ feeling obligated to answer.	Enrollment forms were simplified as follows: Removed questions related to citizenship, eligibility for public benefits, and household income because these were considered sensitive and potentially diminishing the trust in the study. Added questions about language access, including questions regarding comfort with using interpreters, how they navigate language access at the doctor’s office, and understanding health materials. Removed confusing terms such as “cognitive” and “caregiver.” The CAB’s insights led to broader recommendations for more accessible language throughout the form, such as “brain health” and “caring for or providing assistance,” respectively.
**TeleOjo**	**Guidance and feedback:** CAB members played a key role in shaping bilingual materials that explain teleophthalmology services, focusing on accessibility and clarity for Latino communities with elevated risks of diabetes and dementia. Through 3 meetings, CAB members reviewed 1 flyer (including text and images), provided feedback to ensure that the materials were engaging and easy to understand, and prioritized implementation strategies to facilitate access to teleophthalmology.	The Appendix shows the flyer before and after review by the CAB members, which had a few specific changes: Added a definition of retinography Modified the order of the sections, to provide information about the cost and process later in the flyer. Modified language to be easier to read and minimized the use of words that are not commonly used.
**Swallowing in ADRD**	**Relevance and feedback:** The CAB assessed the relevance of the swallowing topic for the Latino/a/e/x senior community. They provided feedback to the speech-language pathologist speaker by asking questions and offering comments and suggestions on how to present the information in community settings. **Outreach and engagement:** advisors participated in community events and invited people to attend and shared resources.	In July 2024, the research team in conjunction with other subject matter experts from the university held the first “Swallowing Talk” for Latino seniors in Madison, in collaboration with the Swallowing and Salivary Bioscience Lab at University of Wisconsin — Madison. The event welcomed 40 Latino older adults and provided transportation and food for all participants. From the swallowing talk, we learned that personal invitations to community members have better reception and are more meaningful compared with broad invitations. The event was open to the entire community, promoted through community partners and social media. Despite these efforts, no participants registered through these channels. All registrations came from personal invitations based on the approach suggested by the CAB members.
**Alzheimer’s disease and related dementia educational tool**	**Guidance and feedback:** Provided feedback on an educational tool to reduce misconceptions about Alzheimer’s disease and related dementia in the Latino community. The CAB reviewed the materials and provided feedback on the feasibility of use, content (attitudes about it and knowledge), language, thoroughness, and complexity.	Feedback from the CAB was used to modify the educational tool. Some modifications included clarifying instructions for community health workers who would be the primary users of the tool, differentiating the terms Alzheimer’s and dementia, and adding a section on early detection and stigma.

To request feedback on specific materials, the CAB members were presented with the materials and the facilitator asked general questions (eg, What do you think? What calls your attention? Is the information easy to understand?), followed by revision of each section of the materials with similar questions (eg, facilitator said, “Let’s look at the first paragraph. What do you think?”). Specific questions were modified according to the project, and we requested comments only on elements that were modifiable. For example, in one of the projects, the wording on the informed consent could not be modified, so this was not presented to the CAB for review. The PAT-1 toolkit provides additional examples of how to collaborate with CAB members in these types of processes.

## Implications of Engaging the Community

Engaging with a CAB extends beyond formal meetings and structured activities; it involves actively participating in initiatives that resonate with the advisors’ interests and the community’s needs. For example, because of the research team’s work with the CAB, they have been contacted by public health agencies and nonprofit service organizations requesting input on upcoming initiatives, have received invitations to synergize efforts, and have received requests to facilitate other community engagement activities that sought to improve the quality and accessibility of services. These activities enhanced the team’s understanding of community needs and demonstrated our commitment to actionable outcomes, strengthening the trust and reciprocity between our research team and the CAB members. This approach ensured that our collaboration extended beyond the confines of research, contributing to tangible community benefits and reinforcing the value of community-engaged research practices.

## Lessons Learned and Recommendations

Considering the principles of community engagement, and based on our experiences, we have outlined recommendations for future community engagement work (Table 2). As the advisors engaged with external investigators, our team established some standards to ensure that the culture and level of the relationship were not affected. One of our first decisions was that all contacts and communications with the CAB members would go through the research team. This allowed us to ensure that these communications were written in plain language and that the CAB members received all the information in a format that they preferred while also minimizing confusion about who was the right person to contact. Additionally, we established the culture of our collaboration and held other investigators to the same standards, rejecting participation in projects that would not commit to these (eg, projects that wanted to use the CAB as research participants rather than advisors or collaborators).

**Table 2:** Recommendations for Practical Implementation of Community Advisory Board (CAB) Engagement Principles with Latino Communities in Madison, Wisconsin

Principle of community engagement and recommendation	Description
**Go to the community, establish relationships, build trust.**	
1. Build trust with partners	Partner with organizations who are already trusted in the community, who can advocate on your behalf, and who can provide credibility and recognition when recruiting advisors. The research team should mirror the diversity of the community at every level (not only the outreach staff). This ensures that the team will have a better understanding of cultural nuances that can impact interactions and outcomes. Furthermore, having multiple people ensures sustainability even if there is staff turnover.
**Be clear about the purposes or goals of the engagement effort.**	
2. Establish clear expectations	During the first meeting, the research team and CAB members should discuss how they want this space to function, set group agreements (eg, communication preferences, meeting preferences), and ensure a shared vision or idea of what success will look like. Ideas should primarily come from CAB members, and the research team can guide conversations and help when needed. Consider using a brainstorming session to encourage dialogue and active participation of all members. Capture ideas in real-time on a whiteboard or shared digital document. This creates a visible record that members can reference and build upon, while also showing that all voices are heard and ideas are thoroughly explored. The individual and group expectations should be reviewed regularly, particularly when reevaluating goals based on achievements and challenges.
**Recognize and respect the diversity of the community in all aspects of community engagement. Awareness of the various cultures of a community and other factors affecting diversity must be paramount in planning, designing, and implementing approaches to engaging a community.**	
3. Ensure inclusive communication	If the community’s preferred language is Spanish: ensure the presence of staff who are native or heritage Spanish speakers, as this ensures smooth conversations without the need for interpretation. When communication occurs in one’s native language, it enhances comfort and reduces the cognitive load associated with translating complex ideas, thereby improving engagement and understanding. Additionally, native speakers are better at recognizing culturally specific nonverbal cues, ensuring more accurate communication and response. Be aware of different cultural preferences or experiences between countries of origin, which is crucial for tailoring messages to ensure relevancy and understanding. Use the community’s preferred forms of communication and provide comprehensive technical assistance to overcome barriers to participation (eg, updating software, troubleshooting any technical issues, providing devices or internet services). Consider tools that advisors are already using in daily communications (eg, WhatsApp, a messaging application widely used by the Hispanic community).
4. Maintain open communication	Use bidirectional channels of communication that provide multiple options for advisors to ask questions or provide suggestions (eg, phone, SMS text message). Ensure that all the advisors understand the different channels available, monitor communications regularly, and understand that there will be different preferences across advisors. Be open to input and show that their opinions are valuable. This promotes an environment where they are not afraid to speak up about the concerns that they might have. Foster an environment of transparency and honesty, where the limitations of the work or administrative or organizational restrictions are shared candidly with a commitment to find solutions within the established parameters.
5. Offer honoraria and recognition	Advisors should be fairly compensated for their time, expertise, and other expenses associated with participation (eg, parking, transportation, childcare). When determining compensation, consider not only meeting time but also preparation time. Learn about barriers that advisors may experience, such as lack of banking access, cash payment needs, or concerns of jeopardizing social security benefits or Medicaid eligibility. Understanding institutional regulations and sharing those with CAB advisors during preliminary stages is essential to explore alternatives. Advisors should be recognized or acknowledged in any manuscript or presentations related to the work.
6. Affirm CAB members’ contributions	Consistently remind the CAB members that their lived experiences and perspectives are invaluable to the research process. Particularly in the Latino community, research has shown that Latino older adults often exhibit a high level of humility and respect for authority figures, including health care providers, which translates into the need for affirming their contributions. This is also key in avoiding power dynamics between the CAB members and the research team. Communicate through concrete examples how the advisors’ input shapes research decisions, influences study designs, and ultimately contributes to community well-being. This ongoing validation boosts advisors’ confidence in sharing their views and enhances their engagement in CAB activities.
7. Understand power dynamics between the academic institution and the community	Agree on a decision-making process. Consider adopting a consensus-based decision-making process, allowing all CAB members to have equal say in discussions and decisions. Use plain and accessible terminology for all communications and presentations to ensure that everyone understands the information that is being presented. Reflect about the meetings as a team, and individually with each CAB member, including what could have been better, or what could be improved. These reflections offer additional opportunities to voice any suggestions or concerns, ensuring continuous improvement and fostering an inclusive and equitable environment.
**Identify and mobilize community assets and strengths and develop the community’s capacity and resources.**	
8. Ensure adequate staff and programmatic support	Ensure that all team members understand the importance of maintaining trust and engagement with the community and are committed to cultural humility and applying the principles of community engagement. This is a key aspect for consideration during the hiring and onboarding of new team members. Ensure an equitable distribution of tasks and prevent overburdening any members. This includes having more than 1 person in the team with the cultural awareness and language skills necessary to perform the work in case of planned or unplanned absences. Consider training and expertise necessary to perform the work and develop training plans to facilitate incorporation of new team members. In the case of changes in roles or staffing, doing a warm handoff of CAB and community members is necessary to foster informal connections and ensure sustainability of the relationships.

## Conclusion

CABs are crucial in fostering strong collaboration between researchers and the community, leading to the creation of culturally relevant and effective solutions. By involving community members who have direct experience with the Latino community, CABs help ensure that research questions, methodologies, and interventions are aligned with real-world needs and cultural nuances. Furthermore, when community members are involved in shaping research and interventions, they are more likely to support and engage with the resulting programs, increasing the likelihood of sustained positive outcomes. This collaborative model not only advances scientific understanding but also ensures that this knowledge translates into meaningful improvements in the quality of life for those affected by dementia.

## Figures and Tables

**Figure:**
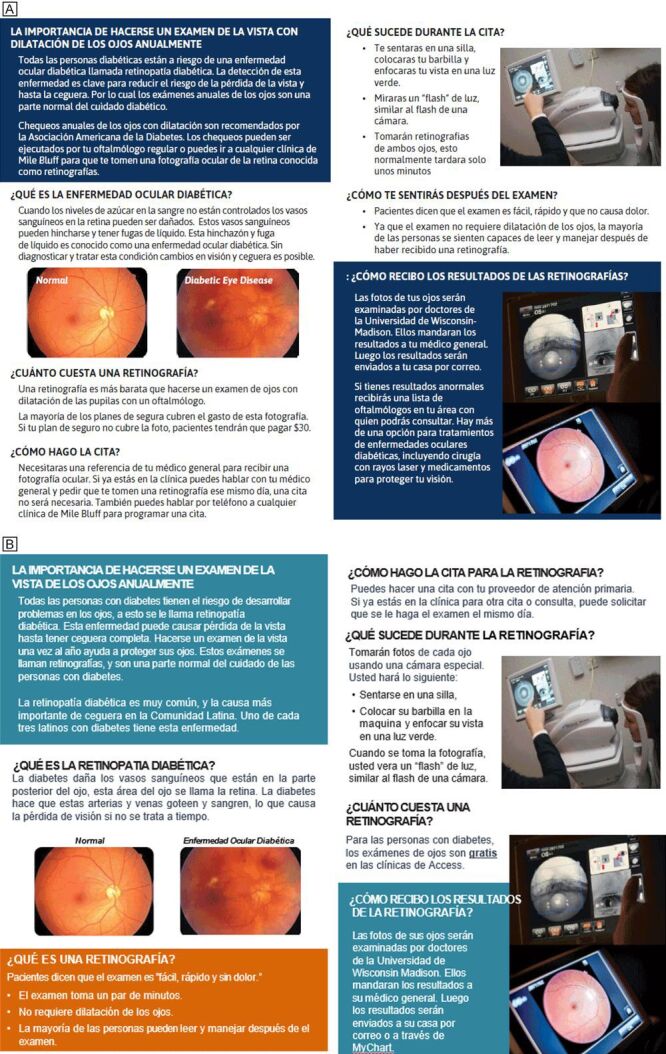

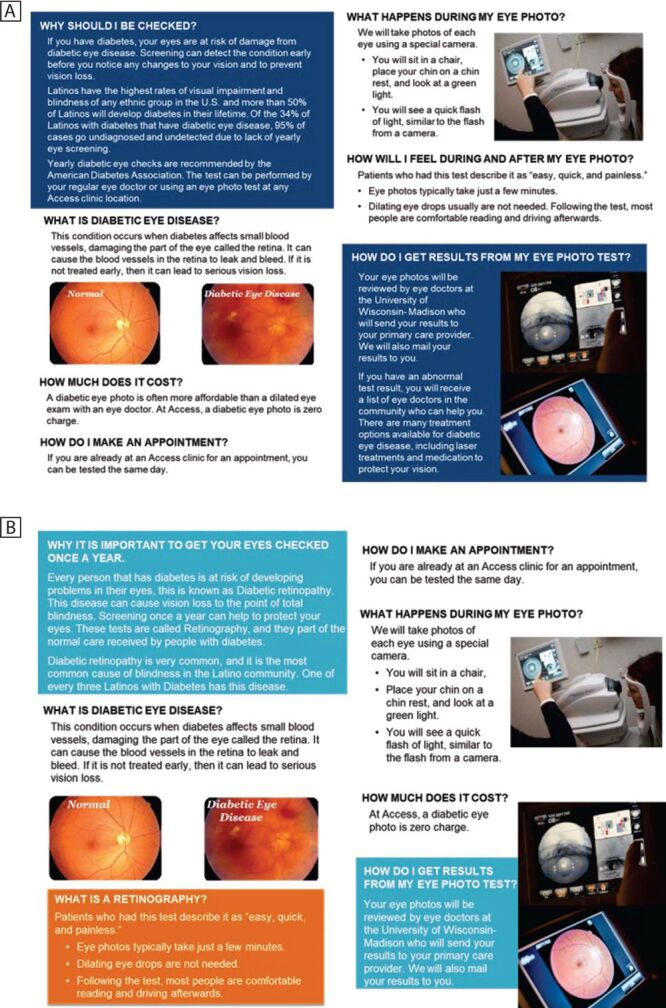
Sample of a flyer in Spanish modified by the community advisory board for project TeleOjo. Panel A is the original flyer, a version of what was already being used in English. Panel B is the flyer after modifications by community advisory board members. The English translation of this flyer appears below the flyer in Spanish.
